# Supplementary material for: Detecting critical nodes in forest landscape networks to reduce wildfire spread
Source: PLoS One. 2021 Oct 7;16(10):e0258060. doi: 10.1371/journal.pone.0258060 (PMC8496796; doi:10.1371/journal.pone.0258060)
Supplement: S1 File — (PDF) [file pone.0258060.s001.pdf]

SUPPLEMENT S1. BURN-P3 MODEL INPUTS

We used the Burn-P3 model (Parisien et al. 2005) to generate stochastic fire ignitions and spread scenarios in our study area. Burn-P3 follows the Canadian Forest Fire Behaviour Prediction (FBP) System, developed by the Canadian Forest Service, to simulate fires and surface fires (Stocks et al. 1989). The critical weather conditions under which the transition from surface to crown fire occurs are dependent on fuel type. While spot fires are not discretely modelled within the FBP System, the empirical rate of spread equations are based on wildfire observation data for high-intensity crown fires, thus effectively incorporating the role of spot fires and ember transport into the rate of spread models.

Burn-P3 uses the Prometheus fire growth model (Tymstra et al. 2010) to simulate the spread of individual fires. The Burn-P3 model repeatedly generates a single stochastic fire season, known as an iteration, which will contain a number of wildfire ignitions based on a draw from the historical fire occurrence distribution. The ignition location and seasonality and day to day fire spread conditions are based on a draw from historical probability distributions. An important and widely used model output is burn probability, which defines the relative likelihood of a fire burning any given pixel.

We parameterized the Burn-P3 with the scenario described in Reimer et al. (2019) for Kootenay National Park. The scenario assumed that fire management crews attack all detected fires in the study area but, on average, 13% of ignited fires would escape initial attack. Fire suppression has the greatest impact on fires which remain small at the time of discovery and less impact on large fires.

Burn-P3 uses probabilistic estimates based on historical fire records to select the number of ignitions per iteration, cause, location, season and fire weather (Parisien et al. 2005). Fuel types and other spatial inputs were based on datasets provided by Parks Canada and the Canadian Forest Service and gridded at a 30-m resolution. The fuel type map included common fuel types from the FBP System (Stocks et al. 1989). The model required spatial data on elevation, fuels, relative probabilities of ignitions by season, wind directions and speed (Table S1). Spatial ignition patterns were based on historical fire records from 1925 to 2015. The model also included human-caused ignitions near roads, campgrounds and human structures (Reimer et al. 2019). Non-spatial inputs included the frequency distributions of escaped fires >1 ha, numbers of spread days per fire and daily fire weather and associated Fire Weather Index parameters grouped by season (Van Wagner 1987). The model also required defining the start and end of the spring and summer periods, fuel green-up dates, and grass curing proportions. We assumed that spring fire weather occurs from April 15 to June 19, and summer weather occurs after June 20. Full description of the model parameterization can be found in Reimer et al. (2019).

Table S1. Burn-P3 input variables.

Name	Data Type	Description
Topography	Raster grid (numeric)	Elevation (m)
Fuels	Raster grid (nominal)	Canadian Forest Fire Behaviour Prediction System fuel types (Forestry Canada Fire Danger Group 1992)
Ignition locations	Raster grids (4 grids, numeric)	Relative probability of ignition by season (spring, summer) and by cause (lightning, human)
Number of ignitions	Frequency distribution (numeric)	The number of escaped fires ≥ 1.0 ha per iteration, range = 1-12 fires
Fire duration	Frequency distribution (numeric)	Number of spread days per fire, in days, range = 1-11 days
Daily fire weather conditions	List of burning conditions (numeric)	Daily fire station weather at noon and associated Fire Weather Index System components (Van Wagner 1987), partitioned by season. Only records with FWI values ≥ 15 were retained
Fire seasons	Setting (nominal)	Dates defining the start and end of the spring period and summer period for selection of fire weather, green-up dates, and grass curing percentage. Spring weather occurs from April 15 to June 19, and summer weather occurs after June 20.
Wind direction	Raster grids (8 grids, numeric)	Influence of topography on wind direction (degrees) for the eight cardinal and ordinal directions
Wind speed	Raster grids (8 grids, numeric)	Influence of topography on wind speed (kmh^{-1}) for the eight cardinal and ordinal directions
Other fire growth parameters	Settings	Fires were allowed a maximum of 3 hours of burning per day, and grass fuel load was set at 3.5 t/ha

REFERENCES:

Forestry Canada Fire Danger Group. Development and structure of the Canadian Forest Fire Behavior Prediction System, Forestry Canada Information Report ST-X-3, Ottawa, ON. 1992.

Parisien, MA, Kafka V, Hirsch KG, Todd JB, Lavoie SG, Maczek PD. Mapping Wildfire Susceptibility with the BURN-P3 Simulation Model; Natural Resources Canada, Canadian Forest Service, Northern Forestry Centre, Information Report NOR-X-405: Edmonton, AB, 2005.

Reimer J, Thompson DK, Povak N. Measuring initial attack suppression effectiveness through burn probability. Forests 2019; 2: 60. doi:10.3390/fire2040060

Stocks BJ, Lynham TJ, Lawson BD, Alexander ME, Wagner CEV, McAlpine RS, Dubé DE. Canadian Forest Fire Danger Rating System: An Overview. For. Chron. 1989; 65: 258-265.

Tymstra C, Bryce RW, Wotton BM, Taylor SW, Armitage OB. Development and Structure of Prometheus: The Canadian Wildland Fire Growth Simulation Model; Natural Resources Canada, Canadian Forest Service, Northern Forestry Centre, Information Report NOR-X-417: Edmonton, AB, 2010.

Van Wagner CE. Development and structure of the Canadian Forest Fire Weather Index System. Canadian Forestry Service, Environment Canada Forestry Technical Report 35; Ottawa, ON, 1987.
