# Supplementary material for: Detecting critical nodes in forest landscape networks to reduce wildfire spread
Source: PLoS One. 2021 Oct 7;16(10):e0258060. doi: 10.1371/journal.pone.0258060 (PMC8496796; doi:10.1371/journal.pone.0258060)
Supplement: S2 File — (PDF) [file pone.0258060.s002.pdf]

SUPPLEMENT S2. CALCULATING THE FIRE SPREAD PROBABILITIES p_{ij}

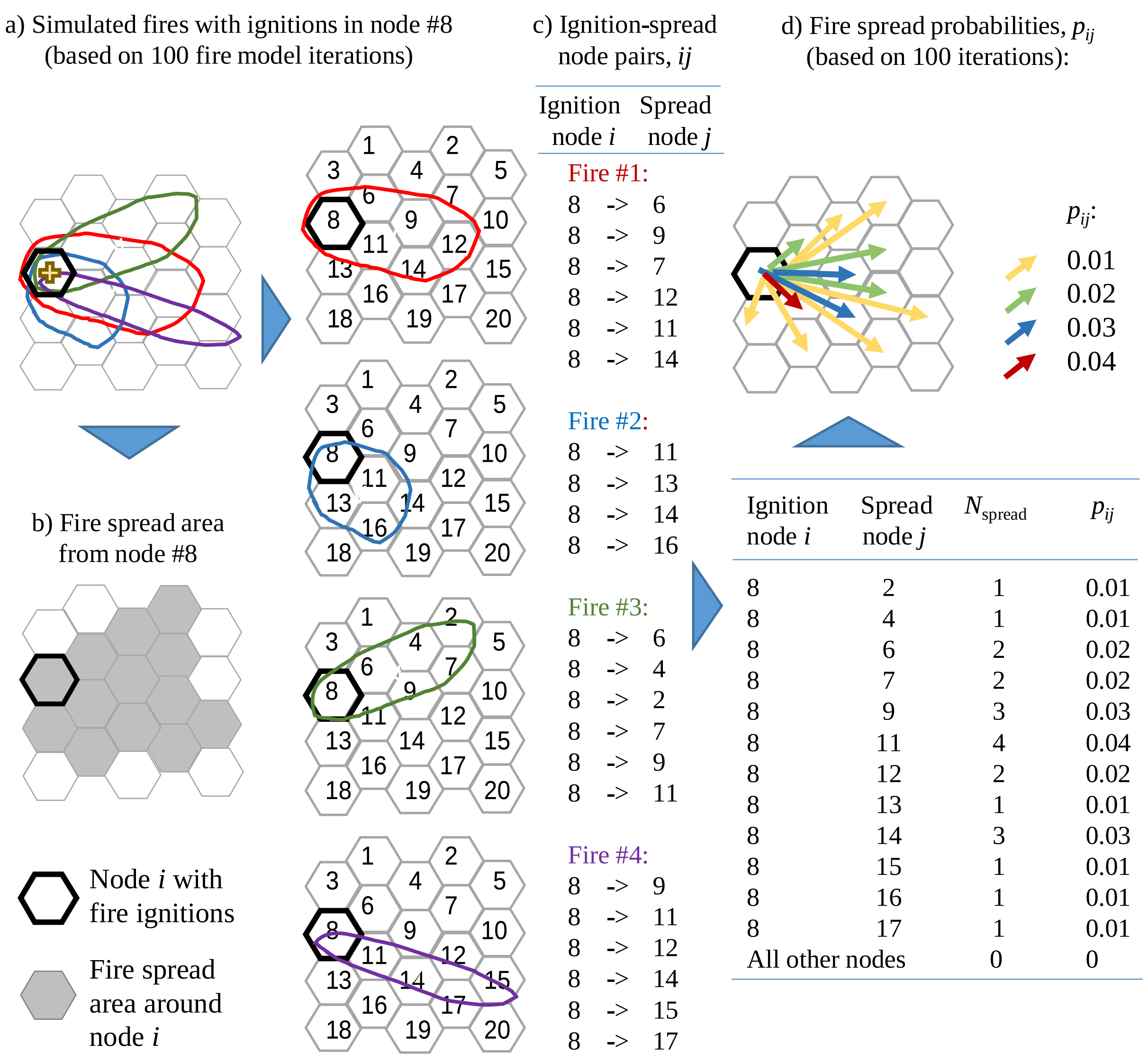

S2 Fig.1. Calculating the fire spread probabilities p_{ij} from node i: a) simulated fire perimeters from ignition locations in node i (node #8); b) fire spread area (fireshed) around node i; c) node pairs ij indicating the ignition locations i and fire spread destinations j; d) fire spread probabilities p_{ij} for node pairs ij.
