# Supplementary material for: Detecting critical nodes in forest landscape networks to reduce wildfire spread
Source: PLoS One. 2021 Oct 7;16(10):e0258060. doi: 10.1371/journal.pone.0258060 (PMC8496796; doi:10.1371/journal.pone.0258060)
Supplement: S3 File — (PDF) [file pone.0258060.s003.pdf]

SUPPLEMENT S3. OPTIMAL SOLUTIONS OF THE CRITICAL NODE DETECTION PROBLEM VS. THE NUMBER OF BURN-P3 ITERATIONS

We examined solutions where the p_{ij} values were estimated using a range of 5,000 to 55,000 Burn-P3 iterations. Node-based burn probability values (the primary Burn-P3 output) stabilized after 10,000 iterations. More iterations were required to stabilize the p_{ij} values. A larger number of iterations produced a larger set of non-zero fire spread probabilities between node pairs, but as the number of iterations increased, nearly all new p_{ij} values depicted rare fire spread events (S3 Fig.1a).

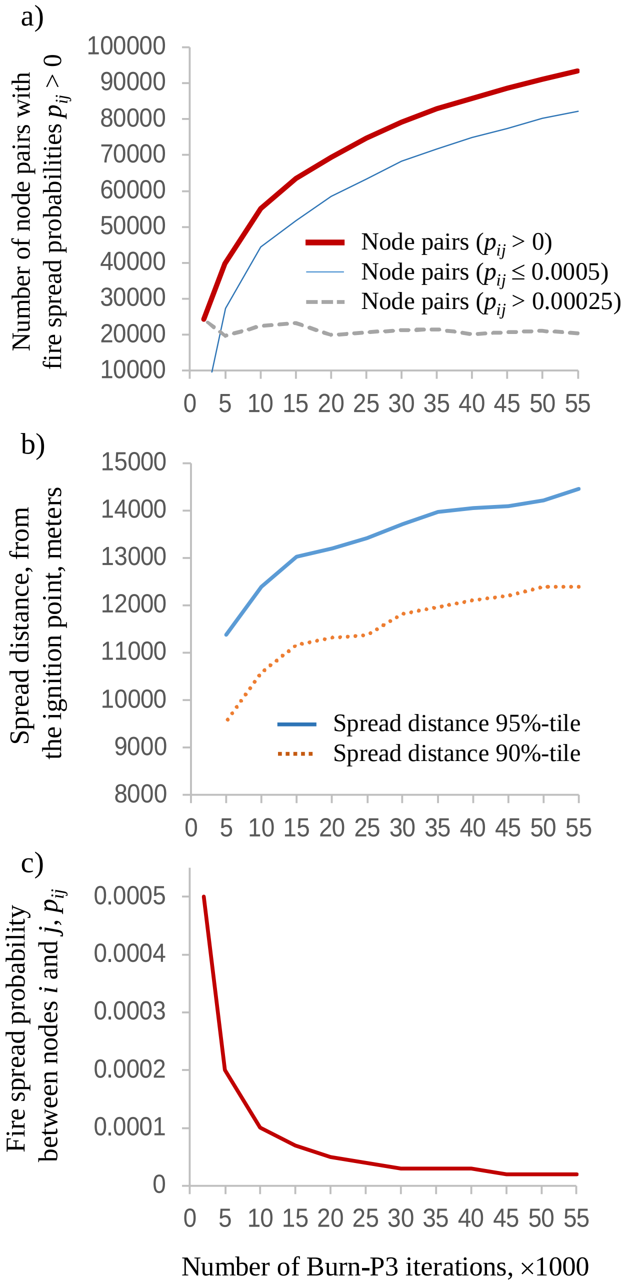

S3 Fig.1. Key fire spread parameters vs. the number of Burn-P3 iterations: a) the number of node pairs *ij* with fire spread probabilities $p_{ij} > 0$, $p_{ij} > 0.00025$ and $p_{ij} \leq 0.0005$ vs. the number of Burn-P3 iterations; b) maximum fire spread distance for 99% and 99.9% of node pairs with fire spread probabilities $p_{ij} > 0$ vs. the number of iterations; c) lowest 5% of the p_{ij} distribution (excluding $p_{ij} = 0$) vs. the number of Burn-P3 iterations.

The number of node pairs with fire spread probabilities above 0.00025 stabilized after 20,000 iterations (S3 Fig.1a). The linear spread distances of 99% and 99.9% of fires started to level off after 50,000 iterations (S3 Fig.1b). The lowest percentile of the p_{ij} distribution stabilized at p_{ij} = 0.0003 after 30,000 iterations (S3 Fig.1c). The node removal patterns in the CND model solutions stabilized after 30,000 iterations (S3 Fig.2), so we report the solutions using p_{ij} values calculated from 30,000 Burn-P3 iterations.

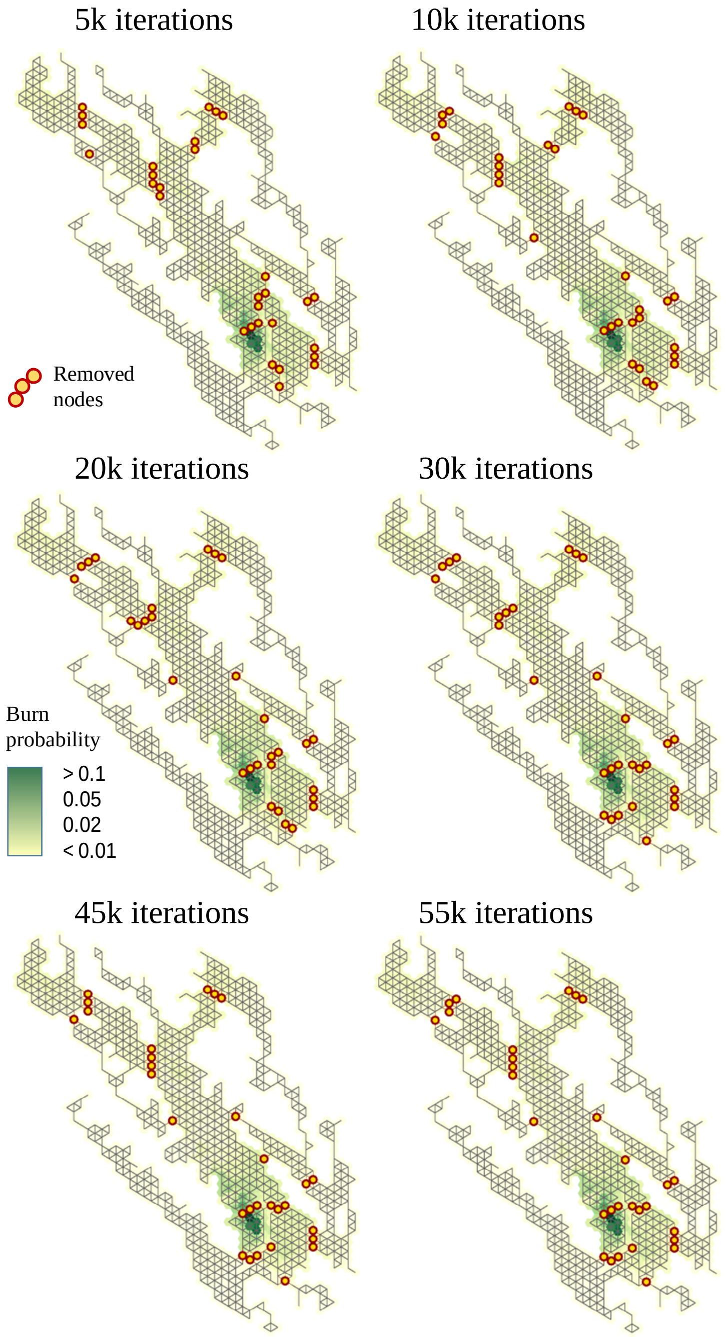

S3 Fig.2. Optimal solutions of the CND model (Equations 2,5-7) vs. the number of Burn-P3 model iterations. Binary fireshed scenario 1, budget B = 30 removed nodes. Large dots show the removed nodes. Gray arcs show edges E in landscape network G. Area shaded in dark green shows the hotspot with highest burn probabilities.
