# Supplementary material for: Detecting critical nodes in forest landscape networks to reduce wildfire spread
Source: PLoS One. 2021 Oct 7;16(10):e0258060. doi: 10.1371/journal.pone.0258060 (PMC8496796; doi:10.1371/journal.pone.0258060)
Supplement: S4 File — (PDF) [file pone.0258060.s004.pdf]

SUPPLEMENT S4. CALCULATING THE FIRE SPREAD PROBABILITIES w_{ij} BETWEEN ADJACENT NODES IN A LANDSCAPE NETWORK

We used the Burn-P3 spatial fire simulation model to calculate fire spread probabilities between adjacent patches in a landscape network of connected forest patches. Similar to other fire simulation models, Burn-P3 generates the locations of ignition points and the perimeters of individual fires simulated for a fire season (Parisien et al., 2005; Riley et al., 2018). Stochastic generation of ignition events driven by fuel composition and weather is followed by generating the perimeters of ignited fires with the Prometheus fire growth model (Tymstra et al., 2010). The Prometheus model is based on the Canadian Fire Behavior Prediction (FBP) system (Stocks et al., 1989). Here we parameterize the Burn-P3 model to repeatedly generate a single stochastic fire season, known as an iteration, as in Parisien et al. (2005).

We calculated w_{ij}, the probabilities of fire spread between adjacent patches (nodes) i and j, from Burn-P3 outputs with ignition locations and simulated fire perimeters. The setting was the hexagonal network G of connected nodes that we used to calculate the fire spread probabilities p_{ij} between all possible node pairs and parameterize the CND model. For each fire generated by Burn-P3, we estimated the fire spread paths from the node with the ignition location to all other nodes in the fire's perimeter area as follows. For all nodes j in the perimeter area, we calculated the shortest paths from node i with the fire's ignition point, $j \neq i$, using Dijkstra's algorithm (Dijkstra, 1959). For each arc ij connecting a pair of *adjacent* nodes in the perimeter area, we incremented the arc value by +1 if ij was a member of at least one shortest path connecting the ignition node and any other node in the perimeter area. The graph of incremented arcs is a union of the shortest paths between the ignition node and all other nodes in the perimeter area (S4 Fig.1a). We repeated this procedure for all fires generated by Burn-P3. The final value of an arc ij connecting adjacent nodes i and j defined the number of times a fire spread from i to j. Dividing this value by the total number of Burn-P3 iterations gave us the probability of fire spread between a pair of *adjacent* nodes i and j, w_{ij}.

We used a similar algorithm to calculate the fire spread probabilities w_{ij} in the scenarios with nodes removed based on the CND model solutions. Recall that the binary decision variables u_{ij} in the CND model indicate the presence of a connecting path between a pair of nodes i and j, and the fire spread probabilities p_{ij} depict the probabilities that a fire ignited in node i will spread to node j. For each fire, the shortest paths were calculated only between node pairs for which the product of the fire spread probabilities p_{ij} and decision variables u_{ij}, $u_{ij}p_{ij}$, was above zero. Factoring in the CND model decision variables u_{ij} accounted for a blocking effect of firebreaks on fire spread in the CND solutions (S4 Fig.1b). After calculating the adjusted paths with firebreaks for all simulated fires, the rest of the w_{ij} calculation was similar to the case without firebreaks. Note that we only used the w_{ij} values to visualize the patterns of fire spread; the optimization model used the actual fire spread probability values p_{ij}.

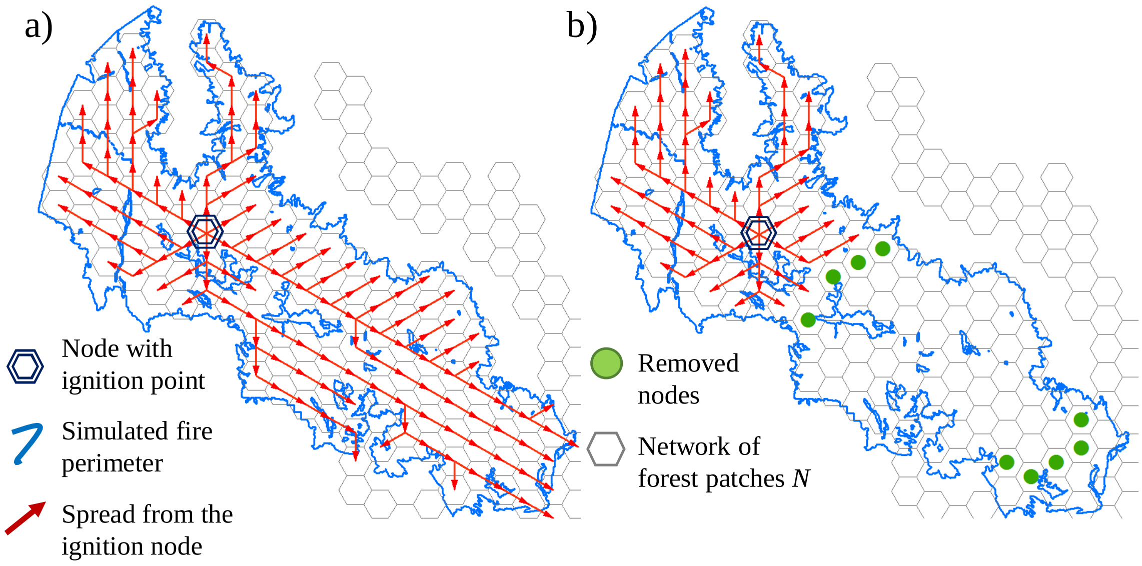

S4 Fig.1. Fire spread paths from the node with the ignition location to other nodes within the fire perimeter: a) fire spread arcs between adjacent nodes in the scenario without fire breaks; b) fire spread arcs between adjacent nodes in the scenario with fire breaks.

The current Burn-P3 version (as of November 2020) can only save the ignition locations and final perimeters of simulated fires. No information is available about the spread of individual fires at intermediate time steps between the time of ignition and extinguishment of a fire. Lack of these intermediate outputs necessitated the use of the shortest path algorithm to interpolate the fire spread paths within individual simulated fires. Shortest path interpolation could be a shortcoming for depicting spread of very large fires, whose local spread directions may deviate from the shortest path due to rapidly changing weather conditions. This is a relatively minor concern for fires in our study area, where fires have a low level of spatial eccentricity (Parisien et al. 2006). True directions of local spread could only be reconstructed from data characterizing the gradual expansion of individual fires over time (such as sequences of individual fire perimeters at intermediate time steps). Adapting the algorithm to utilize such intermediate outputs could be the focus of future work.

REFERENCES:

Dijkstra EW. A note on two problems in connexion with graphs. Numerische Mathematik 1959; 23(3): 269-271. doi:10.1007/BF01386390

Parisien MA, Kafka V, Hirsch KG, Todd JB, Lavoie SG, Maczek PD. Mapping Wildfire Susceptibility with the BURN-P3 Simulation Model; Natural Resources Canada, Canadian Forest Service, Northern Forestry Centre, Information Report NOR-X-405: Edmonton, AB, 2005.

Parisien MA, Peters VS, Wang Y, Little JM, Bosch EM, Stocks BJ. Spatial patterns of forest fires in Canada, 1980–1999. International Journal of Wildland Fire 2006; 15(3): 361-374.

Riley KL, Thompson MP, Scott JH, Gilbertson-Day JW. A model-based framework to evaluate alternative wildfire suppression strategies. Resources 2018; 7: 4.

Stocks BJ, Lynham TJ, Lawson BD, Alexander ME, Wagner CEV, McAlpine RS, Dubé DE. Canadian Forest Fire Danger Rating System: An Overview. For. Chron. 1989; 65: 258-265.
Tymstra C, Bryce RW, Wotton BM, Taylor SW, Armitage OB. Development and Structure of Prometheus: The Canadian Wildland Fire Growth Simulation Model; Natural Resources Canada, Canadian Forest Service, Northern Forestry Centre, Information Report NOR-X-417: Edmonton, AB, 2010.
