# Supplementary material for: Detecting critical nodes in forest landscape networks to reduce wildfire spread
Source: PLoS One. 2021 Oct 7;16(10):e0258060. doi: 10.1371/journal.pone.0258060 (PMC8496796; doi:10.1371/journal.pone.0258060)
Supplement: S5 File — (PDF) [file pone.0258060.s005.pdf]

SUPPLEMENT S5. OPTIMAL SOLUTIONS COMBINING THE NODE REMOVAL STRATEGIES OF THE BINARY AND PROBABILISTIC FIRESHED SCENARIOS

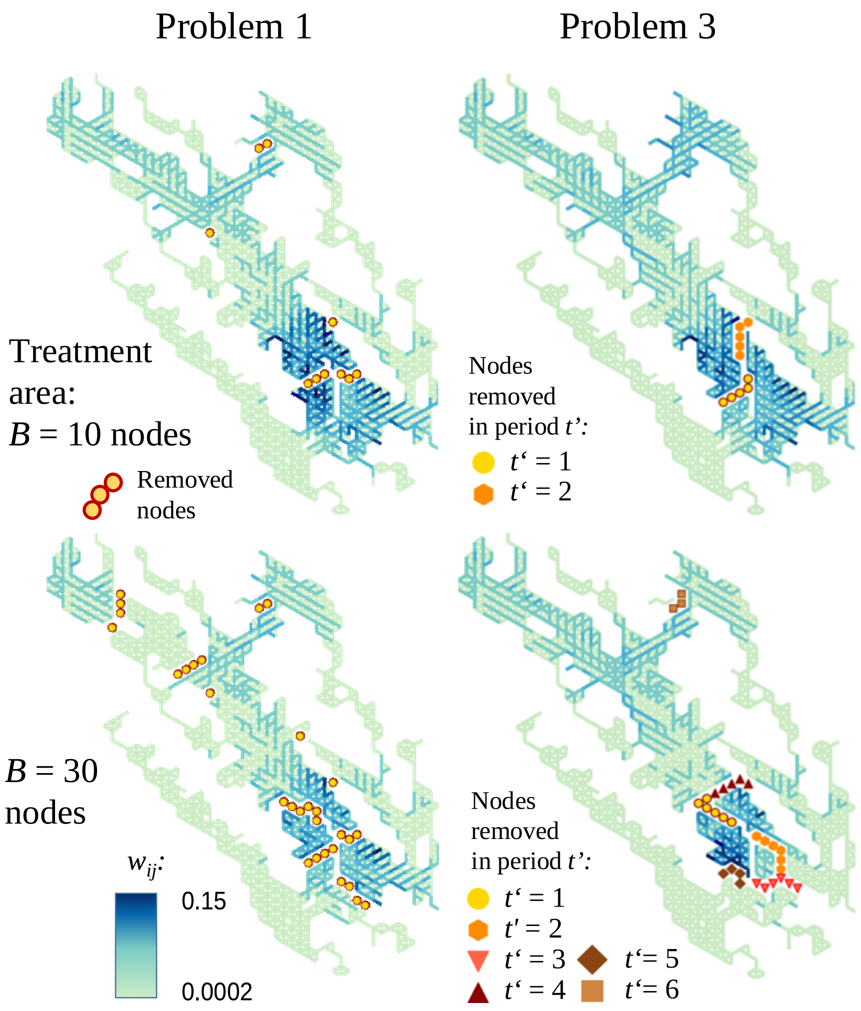

S5 Fig.1. Examples of node removal patterns from solutions in the middle of the trade-off frontiers in Fig.11 in the main text.
