# Supplementary material for: Detecting critical nodes in forest landscape networks to reduce wildfire spread
Source: PLoS One. 2021 Oct 7;16(10):e0258060. doi: 10.1371/journal.pone.0258060 (PMC8496796; doi:10.1371/journal.pone.0258060)
Supplement: Attachment — Submitted filename: response_to_comments_Sept_v2.doc [file pone.0258060.s007.doc]

Manuscript PONE-D-21-15640R1 “DETECTING CRITICAL NODES IN FOREST LANDSCAPE NETWORKS TO REDUCE WILDFIRE SPREAD” – A Reply to Reviewers’ Comments:

Academic Editor:

Lines 326-328, “In general, long-term planning in fire-prone landscapes has little utility because stochastic wildfire activity may override the long-term treatment plans.” is very poor phrasing and inaccurate. Long term planning is a hallmark of forest management generally and there is good work being undertaken at a range of temporal scales that specifically address stochasticity in fire regimes. There is no need for this baseless claim and it should be removed.

We deleted this text as suggested by the Editor.

Reviewer #1:

I am pleased with the revisions. Thank you for clarifying your methods and for explaining where you think the multi-year planning scenario adds value. I still think the method of defining spread probabilities between nodes using the shortest paths from ignition points to perimeters should be framed as an approximation, but this is not central to your work, and the detailed spread paths may not matter much if the “nodes” are large.

We want to clarify that the calculation of fire spread probabilities *p_ij_* between pairs of nodes (which were used in our optimization model) did not involve a shortest path approximation. Recall that the spread probability value *p_ij_* depicts the likelihood that a fire ignited in node *i* spreads to node *j* without indication of how (i.e., by what path) the fire might spread from *i* to *j*. Information about the ignition locations came from the geographic coordinates for individual fires simulated by the Burn-P3 model, and then we used the perimeters of the simulated fires to identify the locations *j* to which a particular fire ignited in *i* could spread.

The only place where we utilized a shortest path approximation was in the calculation of the spread probabilities *w_ij_* between adjacent nodes for visualizing the fire spread patterns. For each simulated fire, we used the shortest path approximation to project the possible fire spread path (with the probability *p_ij_*) between locations *i* and *j* over the network of arcs connecting the adjacent nodes. This map only served to illustrate the fire spread patterns and was not used in optimization modelling. We have edited two sections of the main text, “Calculating the fire spread probabilities *p_ij_*” and “Mapping the fire spread probabilities”, as well as Supplement S4 to make this aspect clearer.

A few minor writing suggestions:

L49-51: I would edit this sentence as: “Our results provide new insights into cost-effective planning to mitigate wildfire risk in forest landscapes. The approach should be applicable to other ecosystems with frequent wildfires.”

Edited as the reviewer suggested.

L64: Drop “in places”?

Dropped.

L78: “fuel treatments” instead of “fuel treatment measures”

Corrected.

L132: Drop “area”

Dropped.

L430-436: Are these sentences necessary after you simplified to an area/node count limit?

These sentences provide background about why we simplified the budget calculations to an area/node count limit and so should stay in the text. Note that the budget constraint [2] would require a variable cost component if the site treatments were solely managed by ground crews, in which case the total treatment cost would depend on the time required to access the treatment sites (which could be a function of complex terrain and proximity to roads).
